# Near-Infrared Markers based on Bacterial Phytochromes with Phycocyanobilin as a Chromophore

**DOI:** 10.3390/ijms20236067

**Published:** 2019-12-02

**Authors:** Olesya V. Stepanenko, Olga V. Stepanenko, Olesya G. Shpironok, Alexander V. Fonin, Irina M. Kuznetsova, Konstantin K. Turoverov

**Affiliations:** 1 Laboratory of Structural Dynamics, Stability and Folding of Proteins, Institute of Cytology, Russian Academy of Sciences, 4 Tikhoretsky ave., St. Petersburg 194064, Russia; lvs@incras.ru (O.V.S.); sov@incras.ru (O.V.S.); olesyashpironok@gmail.com (O.G.S.); alexfonin@incras.ru (A.V.F.); imk@incras.ru (I.M.K.); 2 Peter the Great St. Petersburg Polytechnic University, Polytechnicheskaya str., 29, St. Petersburg 194064, Russia

**Keywords:** bacterial phytochrome, near-infrared biomarkers, iRFP713, fluorescence quantum yield, phycocyanobilin, biliverdin

## Abstract

Biomarkers engineered on the basis of bacterial phytochromes with biliverdin IXα (BV) cofactor as a chromophore are increasingly used in cell biology and biomedicine, since their absorption and fluorescence spectra lie within the so-called optical “transparency window” of biological tissues. However, the quantum yield of BV fluorescence in these biomarkers does not exceed 0.145. The task of generating biomarkers with a higher fluorescence quantum yield remains relevant. To address the problem, we proposed the use of phycocyanobilin (PCB) as a chromophore of biomarkers derived from bacterial phytochromes. In this work, we characterized the complexes of iRFP713 evolved from *Rp*BphP2 and its mutant variants with different location of cysteine residues capable of covalent tetrapyrrole attachment with the PCB cofactor. All analyzed proteins assembled with PCB were shown to have a higher fluorescence quantum yield than the proteins assembled with BV. The iRFP713/V256C and iRFP713/C15S/V256C assembled with PCB have a particularly high quantum yield of 0.5 and 0.45, which exceeds the quantum yield of all currently available near-infrared biomarkers. Moreover, PCB has 4 times greater affinity for iRFP713/V256C and iRFP713/C15S/V256C proteins compared to BV. These data establish iRFP713/V256C and iRFP713/C15S/V256C assembled with the PCB chromophore as promising biomarkers for application in vivo. The analysis of the spectral properties of the tested biomarkers allowed for suggesting that the high-fluorescence quantum yield of the PCB chromophore can be attributed to the lower mobility of the D-ring of PCB compared to BV.

## 1. Introduction

The development of genetically encoded protein fluorescent markers has expanded the possibilities of molecular and cellular biology and biomedicine to study various biological processes in the cell and in vivo. Currently available biomarkers include a group of GFP-like fluorescent proteins of the visible spectral range [1,2,3,4] and a group of near-infrared fluorescent proteins (NIR FPs) derived from photoreceptors with linear tetrapyrroles as ligands. NIR FPs absorb and fluoresce mainly in the region of highest transparency of biological tissues (~650–900 nm). In this spectral range, attenuation of light as a result of absorption by cellular components (hemoglobin, water, lipids, and melanin) and autofluorescence of biological tissues are less pronounced compared to the visible range, and light scattering also decreases with wavelength. Most NIR biomarkers have been developed on the basis of bacterial phytochromes (BphPs) [4,5,6]. The choice of BphPs as a template for NIR biomarkers for application in mammalian, fish, and insect cells is caused by the abundance in eukaryotic cells of the natural prosthetic group of these proteins, biliverdin IXα (BV), which is synthesized as a result of heme catabolism [7]. An analysis of the interaction of NIR FPs designed from BphPs with their BV chromophore have given some insights into the factors affecting the spectral and photophysical properties of these biomarkers [8,9,10,11,12,13,14]. Spectrally distinguishable NIR probes with a fluorescence quantum yield in the range from 0.042 to 0.145 have been developed [6]. The last generation of spectrally distinguishable monomeric NIR biomarkers [15] and NIR biosensors designed on the basis of NIR FPs [16] can be used for labeling individual intracellular proteins and targeting the small signaling molecules and kinase activities, correspondingly. Simultaneous application of these NIR probes with biomarkers and biosensors derived from GFP-like fluorescent proteins and optogenetic tools activated in the blue-green spectral range permits the study of complex cellular processes, in particular cellular signaling.

Phycobiliproteins have been used to evolve BV-binding smURFP (small ultra-red FP) [17] and several BDFPs (BD is used to refer to the Big Dipper constellation) [18], but these probes exhibit lower brightness in mammalian cells compared to BphP-derived NIR FPs. Recently developed NIR biomarkers, termed as biliprotein triads, significantly outperform their parental BDFPs in mammalian cells [19]. The biliprotein triads are composed of three fused BDFPs, in which the excitation energy collected by the covalently attached BV chromophores of the two BDFP domains is transferred to the non-covalently bound phytochromobilin (PΦB) chromophore of the third BDFP domain [19]. Application of biliprotein triads in mammalian cells for labeling intracellular targets requires the supply of the exogenous PΦB. The challenge of extracting PΦB from natural sources has led to the general use of phycocyanobilin cofactor (PCB) instead, which can be isolated easily in large quantities from cyanobacteria [20]. Thus, PCB has been used as a chromophore instead of a natural cofactor for a number of optogenetic tools generated on the basis of plant phytochromes [21,22,23,24]. The optimization of the method for the synthesis of PΦB in the bacterial system has allowed for substantially increasing the yield of this cofactor [19,25]. The BV-binding biomarker miRFP670nano evolved from the chromophore-binding GAF domain of cyanobacteriophytochrome (CBCR) NpR3784, whose natural preference to PCB has been altered by molecular evolution, performs well in mammalian cells with comparable brightness with respect to BphP-derived miRFP670 [26]. Recently discovered CBCRs with uniquely red-shifted absorption maxima of their PCB chromophore compared to other photoreceptors (725–740 nm) can be considered as a promising source for engineering of NIR biomarkers [27].

**Figure 1:**
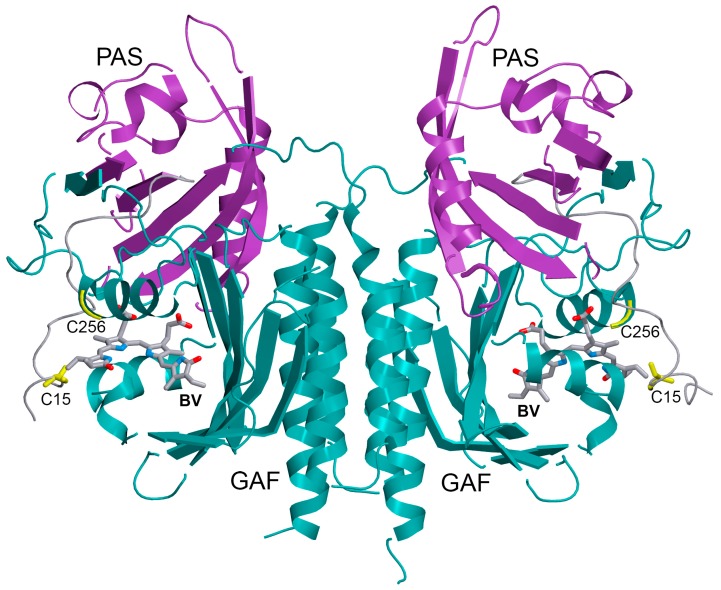
Three-dimensional (3D) structure of the chromophore-binding domain of bacterial phytochrome *Rp*BphP2 (PDB: 4E04 [30]) assembled with its natural ligand, biliverdin IXα (BV). The location of the BV chromophore and cysteine (Cys15)/C256 is shown in sticks. PAS and GAF domains are marked in purple and cyan, respectively. The N-terminal extension of PAS domain is indicated in gray. The drawing was made using the graphic software VMD [31] and Raster3D [32].

Despite significant advances in the molecular evolution of NIR biomarkers, increasing their fluorescence quantum yield and brightness in mammalian cells is highly desirable. To meet this task, we used PCB as a chromophore for iRFP713, developed on the basis of *Rp*BphP2 from *Rhodopseudomonas palustris* [28] (Figure 1). Cyanobacterial and plant phytochromes attach their PCB/PΦB chromophore covalently to a conserved cysteine residue in the GAF domain, while iRFP713 contains a conserved BphP’s cysteine (Cys15) in the N-terminal extension of the PAS domain [29]. In this work, we studied the interaction of PCB with mutant variants of iRFP713: iRFP713/C15S/V256C (the Cys residue was introduced into the conserved region -SPXH- (Cys256) in the GAF domain of the protein), iRFP713/V256C (contains both key Cys residues), and iRFP713/C15S (lacks both key Cys residues).

## 2. Results

### 2.1. The Interaction of iRFP713 and Its Mutant Variants with Phycocyanobilin Cofactor (PCB)

**Figure 2:**
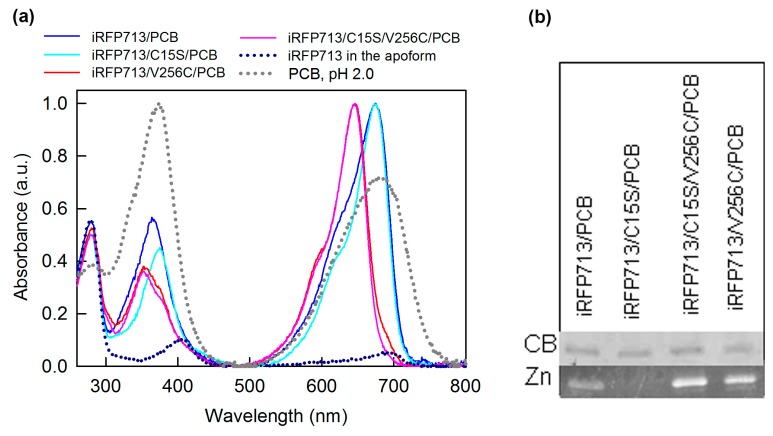
The interaction of the iRFP713 variants with PCB. (**a**) Absorption spectra of the analyzed proteins assembled with cofactor. The spectra of iRFP713 in the apoform (blue dashed line) and of the free BV in protonated form (pH 2.0; gray dashed line) are shown. (**b**) The SDS-PAGE of the PCB-assembled proteins followed by staining with Coomassie blue (CB) and detection of zinc-induced fluorescence of PCB (Zn).

iRFP713 and its mutant variants were capable of binding the PCB ligand. The incorporation of the chromophore into the protein globule was manifested by a change in the absorption spectrum shape of free PCB (position and ratio of the Q and the Soret bands) in the presence of the analyzed proteins (Figure 2a). iRFP713 and its mutant variants chromophorylated with PCB had a high SAR (specific absorbance ratio), which is used to estimate the purity and saturation with a chromophore of phytochromes and cyanobacteriochromes [33,34,35] (Table 1, Figure 2a). This allowed for suggesting that the solutions of the studied proteins assembled with PCB contain a negligible fraction of any cellular proteins or target protein unmodified by the cofactor.

iRFP713/V256C/PCB and iRFP713/C15S/V256C/PCB enclose the covalently attached chromophore, as confirmed by zinc-induced fluorescence of the bands corresponding to the target proteins following separation of protein samples by SDS-PAGE (Figure 2b). The absorption spectrum of iRFP713 in the apoform had absorption bands with maxima at 405 nm and 690 nm (Figure 2a), which indicated the presence of the protein assembled with protoporphyrin IX (PPIX) [36] and BV [28]. PPIX- or BV-bound holoprotein impurities were not detected in the samples of iRFP713/V256C, iRFP713/C15S/V256C, and iRFP713/C15S apoproteins, as evidenced by the absence of absorption bands in the visible spectral region. Since we used the same protocols for purification of iRFP713 in the apoform and in the PCB-bound form, the sample of iRFP713 assembled with PCB is evidently not free of the PPIX- and BV-bound holoproteins. Still, these PPIX- and BV-bound holoprotein impurities contributed little to the zinc-induced fluorescence of iRFP713/PCB (Figure S1). These data argue for a covalent linkage between the PCB ligand and the Cys15 residue in iRFP713 (Figure 2b). iRFP713/C15S, lacking the conserved Cys15 residue, forms a fluorescent complex with PCB in which the chromophore is integrated into the chromophore-binding pocket of the protein but is not covalently linked (Figure 2b).

### 2.2. The Structure of iRFP713 and Its Mutant Variants Assembled with PCB

**Figure 3:**
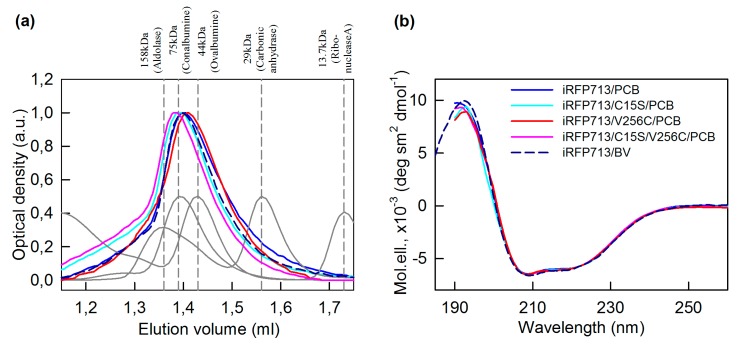
Spatial structure of the iRFP713 variants assembled with PCB. (**a**) Gel filtration of the iRFP713 variants assembled with PCB (the color of the lines is the same as in panel b). The elution profiles of the proteins with known molecular mass used for the column calibration are shown in gray. (**b**) Secondary structure of the cofactor bound iRFP713 variants is tested by far-UV CD. The elution profile on the panel (**a**) and the far-UV CD spectrum on the panel (**b**) of iRFP713/BV are shown as well.

Replacing BV with PCB did not lead to a noticeable change in the spatial structure of iRFP713 and its mutant variants. The elution profiles of iRFP713 and its mutant variants assembled with PCB contained a single peak nearly overlapping the elution peak of the BV-bound iRFP713 holoprotein (Figure 3a). The analyzed proteins assembled with PCB retained the secondary structure inherent to these proteins assembled with their natural ligand, which was proved by CD in the far-UV region (Figure 3b and Table 2). An analysis of intrinsic UV fluorescence of iRFP713 and its mutant variants chromophorylated with PCB revealed a slight increase in the value of parameter *A* (it characterizes the shape and the position of the fluorescence spectra of protein tryptophan residues) compared to the value of parameter *A* of iRFP713 chromophorylated with the natural BV ligand (Table 2). The analyzed proteins assembled either with PCB or BV had almost equal fluorescence anisotropy of tryptophan residues (Table 2). The spectra of tryptophan fluorescence of iRFP713 and its mutant variants chromophorylated with PCB coincided in position with the spectrum of tryptophan fluorescence of iRFP713 with BV as a chromophore (Supplementary Figure S2). The tryptophan fluorescence spectra of the analyzed proteins assembled with PCB and iRFP713/BV normalized to unity at the maximum were superimposed well in the short-wave spectral region but differed in the long-wave spectral edge (Supplementary Figure S2). We have previously shown that an effective energy transfer from the tryptophan residue Trp281 of iRFP713 to BV takes place [37]. Based on this, the observed difference in the tryptophan fluorescence spectra of PCB-bound iRFP713 variants and BV-bound iRFP713 can be explained by the higher efficiency of nonradiative energy transfer from Trp281 to PCB than to BV. The blue shift of the Soret absorption band (the absorption band of tetrapyrroles at about 350–390 nm) of PCB in the studied proteins relative to that of BV in iRFP713 (Supplementary Figure S3) resulted in its increased overlap with the tryptophan fluorescence spectra of the proteins. Thus, the incorporation of PCB instead of BV hardly disturbs the microenvironment of the tryptophan residues of iRFP713 and its mutant variants. Together, the obtained data indicate that the association with different cofactors does not alter the tertiary structure of iRFP713 and its mutant variants.

### 2.3. Spectral Properties of iRFP713 and Its Mutant Variants Assembled with PCB

**Figure 4:**
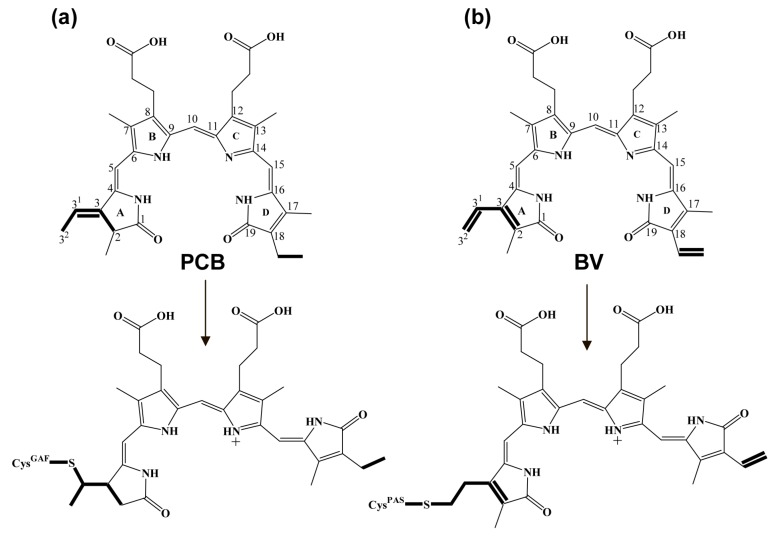
Chemical structure of PCB/BV and their derivatives in photoreceptors. (**a**) The free PCB and the chromophore of cyanobacterial phytochromes and CBCRs. Shown are pyrrole rings A to D with numbered carbon atoms. (**b**) The free BV and the chromophore of bacterial phytochromes (BphPs). Bilins are drawn in 15*Z* configuration. Free bilins are presented in a cyclic conformation, in which they exist in solution [44]. Bilin derivatives are drawn in extended and protonated form, which they assume after incorporation into protein [45]. The differences in the structure of tetrapyrroles and their derivatives are highlighted in bold.

The absorption spectra of iRFP713/V256C/PCB and iRFP713/C15S/V256C/PCB were nearly indistinguishable. A small long-wave shoulder was observed in the Q absorption band (the absorption band of tetrapyrroles in the far-red region) of iRFP713/V256C/PCB (Figure 2a and Table 3). The position of the absorption bands of the PCB chromophore in iRFP713/V256C and iRFP713/C15S/V256C (the Soret and Q-bands are peaked at 353 and 646/647 nm, respectively) was identical to that of cyanobacterial phytochromes [39] and plant phytochromes chromophorylated with PCB [40]. The blue-shifted absorption of PCB assembled with iRFP713/V256C and iRFP713/C15S/V256C relative to the free cofactor is consistent with the loss of one double bond in π conjugation system of the chromophore upon its covalent attachment (Figure 4). By analogy with phytochromes of plants and cyanobacteria, we suggested that the PCB cofactor is covalently conjugated to the Cys256 in the GAF domain of iRFP713/V256C and iRFP713/C15S/V256C via the C3^1^ atom of the A-ring ethylidene.

In contrast, the PCB absorption bands in iRFP713/C15S were red-shifted to 375 and 674 nm, respectively (Figure 2a and Table 3), which is typical for a non-covalently bound cofactor [41,42,43]. Unexpectedly, the position of Q absorption band of the PCB derivative in iRFP713, which is covalently attached to the Cys15 of the protein, coincided with that of the PCB in iRFP713/C15S, which is simply embedded into the pocket of the GAF domain of the protein (Figure 2a and Table 3). Despite a similar position, the absorption spectra of iRFP713/PCB and iRFP713/C15S/PCB differed significantly in the visible region. The short-wave shoulder of the Q absorption band of iRFP713/PCB was more pronounced than that of iRFP713/C15S/PCB. The Soret absorption band of iRFP713/PCB was more intense and blue-shifted relative to the Soret band of iRFP713/C15S/PCB. The BV- and PPIX-bound holoprotein impurities in the samples of iRFP713/PCB are not responsible for the observed spectral differences for iRFP713/PCB and iRFP713/C15S/PCB. 

In bacterial phytochromes, a covalent linkage is formed between the sulfur atom of the Cys residue in the PAS domain and the C3^2^ atom of the A-ring vinyl moiety of the BV cofactor (Figure 4) [46]. An analysis of the interaction of BV with miRFP670 by molecular dynamics methods confirmed the C3^2^ of the A-ring vinyl moiety of the cofactor as the most likely candidate for nucleophilic attack by the sulfur atom of the Cys residue from the N-terminal extension of the PAS domain [47]. The PCB cofactor contains ethylidene side chain at the A-ring with double bond between the C3^1^ and C3 atoms as opposed to the BV cofactor with vinyl side chain (Figure 4). The chemical structure of PCB suggests that only a nucleophilic attack of the C3^1^ atom of the A-ring by the sulfur atom of Cys15 of iRFP713 is possible. The loss of the double bond between the C3^1^ and C3 atoms during the covalent attachment of PCB should result in a short-wave shift of the cofactor absorption bands in iRFP713. However, this was not observed in the case of iRFP713/PCB, implying a certain spectral tuning mechanism in iRFP713/PCB resulting in shift of the chromophore absorption bands toward the longer wavelengths. The bacteriophytochrome AM1_5894 with naturally bound PCB, which was recently isolated from the cyanobacteria *Acaryochloris marina*, demonstrated spectral properties similar to iRFP713/PCB [48]. The absorption spectrum of AM1_5894/PCB in the dark-adapted and light-activated states has a Q-band peak at 682 and 734 nm, respectively. We assumed that the same factors may be involved in the formation of the spectral properties of iRFP713/PCB and AM1_5894/PCB.

**Figure 5:**
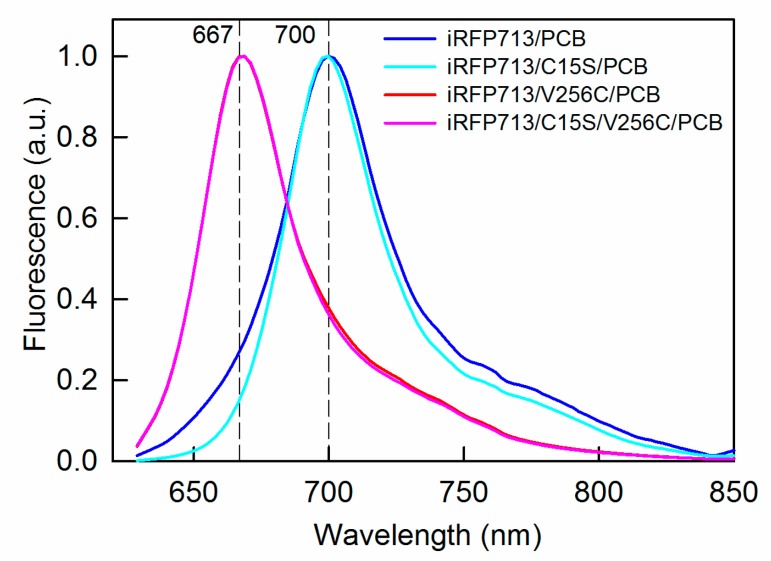
Near-infrared fluorescence of the iRFP713 variants assembled with PCB. The excitation wavelength is 620 nm.

The fluorescence spectra of iRFP713/V256C/PCB and iRFP713/C15S/V256C/PCB coincided well and had maxima at 646 nm (Figure 5 and Table 3). The fluorescence spectra of iRFP713/PCB and iRFP713/C15S/PCB were red-shifted relative to those of iRFP713/V256C/PCB and iRFP713/C15S/V256C/PCB (Figure 5 and Table 3). A slight red shift of 2 nm in the fluorescence spectrum of iRFP713/PCB relative to the fluorescence spectrum of iRFP713/C15S/PCB was detected (Figure 5 and Table 3). The excitation at 560 nm revealed a small shoulder with a maximum at 622 nm in the fluorescence spectrum of iRFP713 (Supplementary Figure S4), which arises from the PPIX-conjugated holoprotein [49].

The fluorescence quantum yield of iRFP713 and its mutant variants assembled with PCB was calculated to be higher than that for the corresponding proteins conjugated with the natural BV ligand (Table 3). The longer excited state lifetime of the PCB chromophore relative to the BV chromophore in analyzed proteins (Table 3) correlates with an increased fluorescence quantum yield of PCB-bond holoproteins [14,50].

### 2.4. A Configuration of PCB Incorporated into iRFP713 and Its Mutant Variants

**Figure 6:**
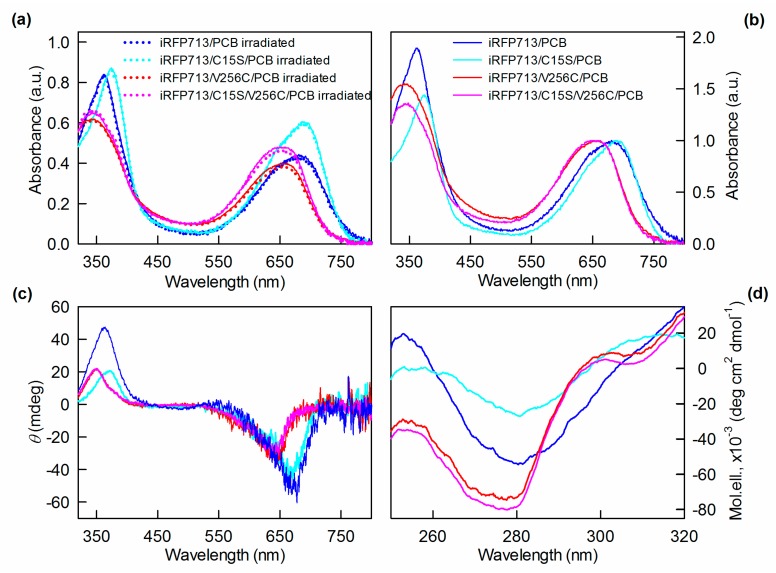
The configuration of PCB incorporated into the iRFP713 variants. (**a**) The absorption spectra of the iRFP713 variants followed the denaturation under acidic conditions. The measurement was performed before (solid lines) and after irradiation with red light (dotted lines). (**b**) The absorption spectra of the iRFP713 variants denatured under acidic conditions normalized to unity at the maximum of the PCB Q absorption band. The visible and near-UV CD spectra of the proteins are shown on the panels (**c**) and (**d**), respectively.

The canonical phytochromes of bacteria, cyanobacteria, and plants, regardless of the type of bound tetrapyrrole and the site of its covalent linkage, photoconvert between two photo-species, a red-light-absorbing (Pr) and far-red-light-absorbing (Pfr), in which the chromophore adopts 15*Z* and 15*E* configuration as a result of rotation of the tetrapyrrole D-ring [29]. The geometry of tetrapyrroles is described by the arrangement of substituents at double (*E*-, *Z*-isomers) and single (*syn*-, *anti*-conformers) bonds of methine bridges between the pyrrole rings (Figure 4) [51]. We used a denaturation assay to estimate the chromophore geometry in iRFP713 and its mutant variants assembled with PCB. The maxima of the Q absorption band of the PCB cofactor in iRFP713, iRFP713/C15S, iRFP713/C15S/V256C, and iRFP713/V256C, which were pre-denatured under acidic conditions, lay at about 680, 685, 652, and 662 nm, respectively (Figure 6a). Irradiation with red light of the denatured proteins did not produce a significant change in their absorption spectra. We reasoned that the chromophore in all proteins tested was locked in a 15*Z* configuration.

Denaturation of iRFP713/PCB did not lead to a significant shift of the Q absorption band of the chromophore. These data testify that the non-covalent interactions of the PCB derivative with its microenvironment in iRFP713 are not relevant for the spectral properties of the native complex. For example, the π-stacking interactions of the BV chromophore with the aromatic radicals of phenylalanine in miRFP709 were shown to account for a red shift of the absorption and fluorescence spectra of this protein compared to miRFP703 [52]. It is believed that the π-stacking interactions of the PCB ligand and the indole ring of the tryptophan residue in its microenvironment are responsible for the spectral properties of the core-membrane linker L_CM_, ApcE (the name is given according to the gene by which it is encoded), which is a part of the cyanobacteria phycobilisome [53]. Thus, the chemical structure and chromophore geometry are left as factors underlying the spectral properties of iRFP713/PCB. The stabilization of the 15*E* isomer of PCB in iRFP713 was excluded as a reason for the unique spectral properties of the complex.

The visible absorption spectra of iRFP713/PCB and iRFP713/C15S/PCB, measured under denaturing conditions, differed in shape (Figure 6b). The short-wave shift of the Q absorption band of the denatured iRFP713/PCB compared to the Q absorption band of the denatured iRFP713/C15S/PCB may be due to a less extended π-conjugation system of the PCB-Cys15 relative to that of the free PCB. On the other hand, the denatured iRFP713/PCB demonstrated a higher R/B ratio (the ratio of absorbance in the Soret band to absorbance in the Q-band) compared to other iRFP713 variants assembled with PCB in the chemically denatured state. This may indicate that the PCB-Cys15 in solution adopts a more cyclic conformation than the free PCB and the PCB-Cys256. The short-wave shift of the Q absorption band of the cyclic forms of tetrapyrroles compared to the Q absorption band of their extended forms can be attributed to a less efficient conjugation of the chromophore π-bond system [54].

The absorption spectra of iRFP713/C15S/V256C/PCB and iRFP713/V256C/PCB under denaturing conditions were also different (Figure 6b). Despite the nearly complete overlapping of the Q absorption band of these holoproteins, a slight long-wave shift of this band of iRFP713/V256C/PCB relative to that of iRFP713/C15S/V256C/PCB can be observed. The Soret absorption band was more pronounced for denatured iRFP713/V256C/PCB than for denatured iRFP713/V256C/PCB. Additionally to the Cys256 residue, the iRFP713/V256C protein contains Cys15, which can be involved in covalent binding of PCB. The spectral differences observed for iRFP713/C15S/V256C/PCB and iRFP713/V256C/PCB under denaturing conditions can be explained in the frame of the assumption that PCB managed to bind to Cys15 rather than to Cys256 in some molecules of the iRFP713/V256C. The assumption is in line with the lower fluorescence quantum yield of iRFP713/V256C/PCB compared to iRFP713/C15S/V256C/PCB (Table 3).

Circular dichroism spectroscopy is a sensitive method to assess the chromophore geometry of phytochromes. The Pr states of phytochromes of different origin associated with different tetrapyrrole have a characteristic CD spectrum in the visible region with a strong negative rotational strength in the Q absorption band and a positive rotational strength in the Soret absorption band of the cofactor [45,55,56]. The shape of the CD spectra in the visible region of PCB-assembled iRFP713 and its mutant variants under native conditions supported the 15*Z* configuration of their chromophores (Figure 6c).

iRFP713 and its mutant variants assembled with PCB had pronounced CD spectra in the near-UV region (Figure 6d). Since iRFP713 and its mutant variants demonstrated negligible optical activity in the near-UV region, the optical activity of the PCB-bound holoproteins is determined by the chromophore and by the interaction of cofactor pyrrole rings with protein residues in its vicinity. The near-UV CD spectra of the iRFP713/V256C/PCB and iRFP713/C15S/V256C/PCB were nearly identical. This means that iRFP713/V256C/PCB and iRFP713/C15S/V256C/PCB incorporate the same dominant derivative of PCB. The near-UV CD spectra of both iRFP713/PCB and iRFP713/C15S/PCB were significantly different from the spectra of iRFP713/V256C/PCB and iRFP713/C15S/V256C/PCB. The CD spectrum in the near-UV region of iRFP713/PCB had a shoulder at the wavelength above 290 nm, which was absent in the CD spectrum of iRFP713/C15S/PCB. The data points at the difference in interaction between the PCB chromophore and residues of its microenvironment in iRFP713, iRFP713/C15S, and iRFP713/V256C (iRFP713/C15S/V256C). We assume that the geometry of the PCB derivatives, namely PCB-Cys15, PCB, and PCB-Cys256, in iRFP713, iRFP713/C15S, and iRFP713/V256C (iRFP713/C15S/V256C) varies.

Moreover, the shape of near-UV CD spectra of the analyzed protein was sensitive to the type of bound tetrapyrrole (Supplementary Figure S5), implying the difference in interaction with the protein environment in pairs of PCB-Cys15 and BV-Cys15, PCB-Cys256 and BV-Cys256, and non-covalently bound PCB and BV, respectively.

### 2.5. Competitive Binding of PCB and BV with iRFP713 and Its Variants

Since iRFP713 and its mutant variants assembled with PCB had a high fluorescence quantum yield, these biomarkers can be considered for cell labeling in vivo. However, their application in mammalian cells may be complicated by the presence of endogenous BV. We determined the binding specificity of both cofactors to proteins studied here using the previously developed approach based on the analysis of the competitive interaction of PCB and BV with phytochrome apoproteins [43].

**Figure 7:**
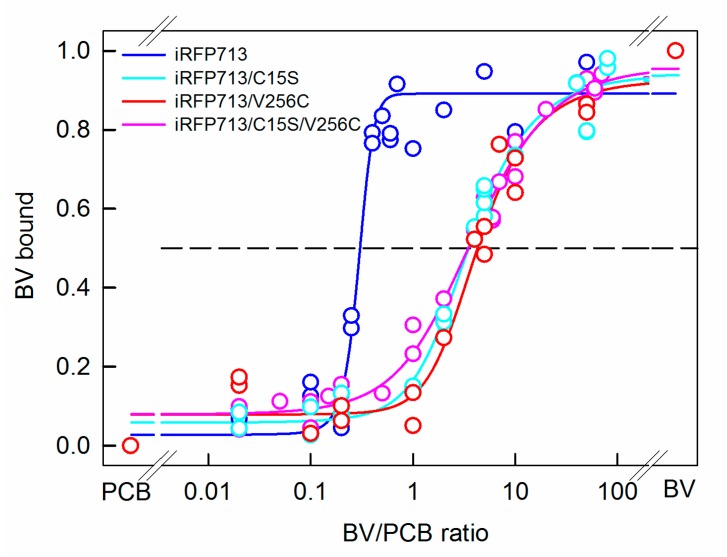
Competing PCB and BV for apoproteins of iRFP713 and its mutant variants. The contribution of BV and PCB assembled with the analyzed proteins to their absorption is determined as described in the “Materials and Methods” section. The dashed line indicates the 50% saturation of the analyzed proteins with BV. The experimental data and their approximation are shown by circles and solid lines, respectively.

The PCB ligand exhibited an approximately 4-fold greater affinity of binding to iRFP713/V256C and iRFP713/C15S/V256C relative to BV (Figure 7). Expectedly, BV bound to iRFP713, bearing Cys residues conserved for BphPs in the PAS domains, four times more efficiently than PCB did (Figure 7). This finding explains the presence in the isolations of the iRFP713 apoprotein of the fraction of BV-bound holoprotein. The preferential binding of BV to iRFP713 was abolished with the C15S substitution. The binding affinity of BV ligand to iRFP713/C15S, lacking the cysteine residues for covalent attachment of a cofactor, was about four times less than that of PCB (Figure 7). The data obtained for iRFP713, iRFP713/C15S, and iRFP713/C15S/V256C are consistent with the data for the corresponding mutant variants of *Dr*BphP [43]. The specificity of tetrapyrroles to iRFP713/V256C and *Dr*BphP/M259C containing two sites for the chromophore covalent linkage varies. In contrast to iRFP713/V256C, which was preferentially targeted by PCB, both ligands could bind to *Dr*CBD/M259C with the same efficiency [43].

## 3. Discussion

We have shown that the use of PCB as a chromophore in bacterial phytochromes instead of their natural BV ligand is an effective way of increasing the fluorescence quantum yield of BphP-derived NIR biomarkers. Indeed, PCB chromophore conjugated to iRFP713 and its mutant variants with different sites of covalent binding of tetrapyrrole has a higher fluorescence quantum yield compared to BV in the corresponding proteins. The fluorescence quantum yield of iRFP713/V256C and iRFP713/C15S/V256C assembled with PCB significantly exceeds the fluorescence quantum yield of all currently available NIR biomarkers. Previous studies indicate that the assembly of full-length bacterial phytochrome *Dr*BphP and a number of its mutant variants with PCB impaired the photoconversion in holoproteins [43]. It was also found that the replacement of the natural PΦB ligand in the plant phytochrome PhyB with PCB affected the Pfr- to Pr-thermal reversion kinetics of the protein [40]. These data emphasize that even a minimal change in the chemical structure of tetrapyrrole has a profound effect on its interaction with phytochrome. This assumption is consistent with our data on CD spectra in the near-UV region of the analyzed proteins chromophorylated with PCB or BV. Nonradiative deactivation of the tetrapyrrole excitation energy in phytochromes occurs mainly via the isomerization of the chromophore around the C15 = C16 bond and the transient proton transfer from the cofactor to the bound pyrrole water or the carbonyl oxygen of the main chain of the residue at position 204 (which is equivalent to the Asp residue of wild-type BphPs in the conserved motif -P_201_XS***D***IP_206_- of the GAF domain) [8,57]. The truncation of full-length BphPs up to the PAS-GAF module and/or mutating of the conserved Asp204 residue mostly blocks productive photoconversion to the Pfr state, but photoproduct states Lumi-R and even Meta-R can be formed under photoexcitation [36,58]. The increased excited-state lifetime of PCB in the analyzed proteins compared to that of BV (Table 3) is indicative of a more efficient competition of radiative pathways than nonradiative ones [11,59]. Activation of both nonradiative channels depends on twisting around the C15 = C16 bond of a tetrapyrrole [14]. This suggests that the limitation of the mobility of the D-ring of the chromophore underlies the increase in the fluorescence quantum yield of PCB compared to BV in the analyzed proteins. We cannot exclude the reorganization of the hydrogen bond network around the PCB chromophore with respect to the BV chromophore in the analyzed proteins that would affect an excited-state transfer of the pyrrole proton. Indeed, the near-UV CD spectra indicate a change of the microenvironment of PCB derivatives relative to the microenvironment of corresponding BV derivatives.

We have previously revealed the allosteric influence of monomers in dimeric NIR FPs on each other [12]. In dimeric NIR FPs, that contain Cys residues in GAF domains but not in PAS domains (including iRFP713/C15S/V256C), covalent attachment of BV to Cys256 in one monomer of the protein allosterically inhibited the formation of a covalent bond between Cys256 and the chromophore incorporated into the GAF pocket of the second protein monomer. Here, we showed that spectral properties of iRFP713/C15S/V256C and iRFP713/V256C assembled with PCB are determined mainly by a ligand covalently linked via the C3^1^ atom of the A-ring ethylidene moiety to Cys256 in the GAF domain of the proteins. The absence of non-covalently bound PCB in the iRFP713/C15S/V256C assembled with this tetrapyrrole may contribute to the high-fluorescence quantum yield of the complex. These data also imply that the interaction between PCB and iRFP713/C15S/V256C, in contrast to the interaction between BV and the protein, is not sensitive to allosteric communication between monomers of the protein.

In conclusion, the preferred binding of PCB, rather than BV, to the iRFP713/V256C and iRFP713/C15S/V256C and the high-fluorescence quantum yield of PCB in the obtained complexes, make them promising biomarkers for use in vivo.

## 4. Materials and Methods

### 4.1. Protein Expression and Purification

The iRFP713 genes were amplified and cloned into a pBAD/His-B vector (Invitrogen, Carlsbad, CA, USA) using BglII and EcoRI sites [60]. For expression of iRFP713 and its variants in the apoforms, LMG194 host cells (Invitrogen, Carlsbad, CA, USA) were transformed by pBAD/His-B plasmid encoding target proteins with polyhistidine tags on the N-termini. Bacterial cells were grown in RM medium (1Х М9 salts, 2 % casamino acids, 1 mМ MgCl2, 1 mМ thiamine) supplemented with ampicillin. The overnight LMG194 culture was grown for 2–3 h at 37 °C, then, protein synthesis was induced by 0.002% arabinose followed by the incubation of cell culture for 12 h at 37 °C and for 24 h at 18 °C. Apoproteins were purified with sequential affinity chromatography on a His Gravitrap column (GE Healthcare, Chicago, IL, USA) and ion-exchange chromatography on a MonoQ column (GE Healthcare, Chicago, IL, USA).

The iRFP713 and its variants were chromophorylated with PCB in vitro. To this aim, the solutions enriched in target apoproteins collected after purification of crude cellular extracts with affinity chromatography were incubated with a 2-fold molar excess of PCB for 3 h at 4 °C in the dark. The reaction was carried out in TE buffer: 20 mM Tris (tris(hydroxymethyl)aminomethane)/HCl, 1 mM EDTA (ethylenediaminetetraacetic acid) (pH 8.0), and 1 mM DTT (dithiothreitol). The concentration of a methanol stock solution of PCB (Frontier Scientific, West Logan, UT, USA) was calculated using an extinction coefficient at 662 nm of 35,500 M^−1^·cm^−1^ in acidic urea (8 M, pH 2.0) [61]. The complexes of iRFP713 and its variants with PCB were then subjected to ion-exchange chromatography.

The purity of the proteins was tested by gel electrophoresis under denaturing conditions (SDS-PAGE) in 12% polyacrylamide gels [62]. The homogeneity of proteins chromophorylated with PCB was evaluated by the value of specific absorption ratio (SAR), which was calculated as the ratio of absorbance at maxima of the Q-band of a chromophore (at far-red/near-infrared spectral region) to absorbance of aromatic resides at 280 nm [33,34,35]. The protein was concentrated and stored in 20 mM Tris/HCl buffer, 150 mM NaCl, pH 8.0.

### 4.2. Spectral and Biochemical Characterization of Proteins

The samples of complexes of iRFP713 and its variants with PCB for spectroscopic characterization were prepared in 20 mM Tris/HCl buffer, pH 8.0. The concentration of the protein samples did not exceed 0.2 mg/mL. All the measurements we carried out at room temperature.

Absorption spectra were acquired using a U-3900H spectrophotometer (Hitachi, Tokyo, Japan) with microcells 101.016-QS 5 × 5 mm (Hellma, Jena, Germany). The fluorescence spectra and parameters were measured using a Cary Eclipse spectrofluorimeter (Agilent, Santa Clara, CA, USA) with cells 10 × 10 × 4 mm with a path length of 10 mm (Starna, Atascadero, CA, USA, USA). The tryptophan fluorescence of the protein was excited at the long-wave absorption spectrum edge (λ_ex_ = 295 nm) to minimize the contribution of the tyrosine residues in the bulk protein fluorescence. The position and form of the fluorescence spectra were characterized on the basis of parameter *A* = *I*_320_/*I*_365_, where *I*_320_ and *I*_365_ are the fluorescence intensities at the emission wavelengths of 320 and 365 nm, respectively [63]. The value for parameter *A* was corrected for the instrument sensitivity. The anisotropy of tryptophan fluorescence was calculated using the equation:

(1) $$r=\frac{\left(I_{v}^{v}-GI_{H}^{V}\right)}{\left(I_{V}^{V}+2GI_{H}^{V}\right)}$$

where $I_{V}^{V}$ and $I_{H}^{V}$ are vertical and horizontal components of the fluorescence intensity excited by vertically polarized light respectively, and $G=I_{V}^{H}/I_{H}^{H}$ is the coefficient that determines the different instrument sensitivity for the vertical and horizontal components of the fluorescence light, λ_em_ = 365 nm [64].

The specific near-infrared fluorescence of iRFP713 and its variants chromophorylated with PCB was excited at the short-wave absorption spectrum edge of the Q-band of the chromophore absorption (560–600 nm).

The extinction coefficient of the complexes of iRFP713 and its variants with PCB was quantified by measuring the absorbance of denatured protein in acidic urea (8 M, pH 2.0) and using the known extinction coefficient of PCB under these conditions [61]. The fluorescence quantum yield of the complexes of iRFP713 and its variants with PCB was determined using iRFP713 and iRFP713/V256C chromophorylated with BV as standards.

The covalent attachment of PCB to iRFP713 and its variants was analyzed by zinc-induced fluorescence after incubating SDS-PAGE gels in 1 mM ZnCl_2_ for 30 min and subsequent staining with Coomassie Blue.

The efficiency of binding PCB relative to BV with iRFP713 and its variants was estimated according to the previously proposed approach [43]. iRFP713 and its variants were incubated overnight in the presence of both tetrapyrroles in a BV to PCB molar ratio of 0.02 to 80 in TES buffer. The unbound chromophores were removed, and the buffer was exchanged with 20 mM Tris/HCl buffer, pH 8.0, using VivaSpin concentrators with 30 kDa molecular weight cut-off (Sartorius AG, Goettingen, Germany). UV-Vis absorption spectra of the solution containing the mixture of a protein chromophorylated ether with PCB or BV were measured. The contribution to the recorded spectra of a protein complex with BV relative to a protein complex with PCB was determined from the ratio:

(2) $$\left(Q_{BV}/Q_{PCB}\right)/\left(Soret_{BV}/Soret_{PCB}\right)$$

where $Q_{X}$ and $Soret_{X}$ are the absorption in the maxima of Q and Soret (at around 350–390 nm) bands of a protein bound to BV or PCB. The calculated values were corrected by subtracting the value for the protein samples bound to PCB and normalized to unity for the protein samples bound to BV. The experimental data were approximated according to the model of competitive ligand binding using Sigma Plot 12.5 software package.

Chromophore fluorescence decay curves of the complexes of iRFP713 and its variants with PCB were acquired using a spectrometer FluoTime 300 (PicoQuant, Berlin, Germany) with the laser diode head (LDH)-C-375 (λ_ex_ = 375 nm) or LDH-C-660 (λex = 660 nm). The fluorescence decay curves were fit to a multiexponential function using the standard convolute-and-compare nonlinear least-squares procedure [65]. Minimization was performed according to Marquardt [66].

### 4.3. Analysis of the Chromophore Geometry

The chromophore geometry in the complexes of iRFP713 and its variants with PCB was analyzed using the denaturation assay [67]. The approach is based on the fact that the 15*Z* and 15*E* isomers of tetrapyrroles have characteristic absorption spectra under denaturing conditions and the later undergoes unidirectional photoisomerization to the 15*Z* conformer after irradiation with red light. The 15*Z* isomer of PCB has absorption spectra with a Q-band peak at about 665 nm, while its 15*E* isomer has absorption spectra with a Q-band peak at about 595 nm. UV-Vis absorption spectra of the denatured protein complexes were measured immediately after the mixing of the protein samples in quenching buffer (0.1% trichloroacetic acid in methanol) and after irradiation of denatured probes for 10 min using 640 nm light-emitting diode lights with a photon flux density of 33 μmol m^−2^·s^−1^.

### 4.4. Gel Filtration Experiments

Size exclusion chromatography of iRFP713 and its variants assembled with PCB were performed at 23 °C using an AKTApurifier system (GE Healthcare, Chicago, IL, USA) on a Superose 12 PC 3.2/30 column (GE Healthcare, Chicago, IL, USA) pre-equilibrated in 50 mM NaH_2_PO_4_ and 150 mM NaCl (pH 8.0). The column was calibrated using a set of proteins with known molecular mass (chromatography standards from GE Healthcare, Chicago, IL, USA). The concentration of protein samples loaded on the column (10 μL) was 0.5 mg/mL. The elution of tested proteins was monitored by absorbance at 280 nm and at the maximum of the Q-band of the PCB or BV chromophore.

### 4.5. Circular Dichroism Measurements

The Jasco-810 spectropolarimeter (Jasco, Tokyo, Japan) was used for the measurement of circular dichroism (CD) spectra. The far-UV (in the range of 260–190 nm), the near-UV (in the range of 320–250 nm), and the visible CD spectra (in the range of 810–320 nm) were recorded using 1 mm and 10 mm path length cells. Three scans of CD spectra were collected, averaged, and corrected by buffer solution background for every probe. The concentration of protein samples was 0.5 mg/mL. The experimental data, recorded in the far-UV and near-UV region of the spectrum, were converted into units of the molar ellipticity per amino acid residue and into units of the molar ellipticity, respectively. The visible CD spectra are represented in units of the ellipticity.

## Figures and Tables

**Table 1:** Spectral characteristics of iRFP713 and its mutant variants assembled with phycocyanobilin cofactor (PCB) *in vitro*.

Protein	Parameter *A* (λ_ex_ = 295 nm)	Fl. Anisotropy r (λ_ex_ = 295 nm, λ_em_ = 365 nm)	SAR ^1^
iRFP713/BV	1.56 ± 0.02	0.125 ± 0.005	1.9
iRFP713/PCB	1.73 ± 0.03	0.125 ± 0.005	1.8
iRFP713/C15S/PCB	1.70 ± 0.02	0.11 ± 0.01	2.0
iRFP713/V256C/PCB	1.65 ± 0.03	0.125 ± 0.005	1.9
iRFP713/C15S/V256C/PCB	1.64 ± 0.02	0.12 ± 0.01	2.0

^1^ Specific absorbance ratio. It is determined as indicated in the “Material and method’ section.

**Table 2:** The evaluation of secondary structure content of iRFP713 and its mutant variants assembled with PCB in vitro.

Protein	Helix ^1^	β-Sheet	β-Turn	Random
iRFP713/BV	0.19	0.31	0.20	0.29
iRFP713/PCB	0.19	0.31	0.21	0.29
iRFP713/C15S/PCB	0.18	0.30	0.21	0.31
iRFP713/V256C/PCB	0.18	0.31	0.21	0.31
iRFP713/C15S/V256C/PCB	0.18	0.30	0.22	0.30

^1^ Analysis was made on the basis of the far-UV CD spectra using the Provencher’s algorithm [38].

**Table 3:** Spectral characteristics of PCB derivatives in iRFP713 and its mutant variants.

Protein	Absorbance Maximum (nm)	Extinction Coefficient at the Main Peak (M^−1^·cm^−1^)	Emission Maximum (nm)	Quantum Yield(%)	Chromophore Fluorescence Lifetime (ns)
iRFP713/BV	692 ± 1	98,000	713 ± 1	6.3 ^1^	0.67 ± 0.01
iRFP713/C15S/BV	685 ± 1	68,000	710 ± 1	5.5 ± 0.3	0.87 ± 0.01
iRFP713/V256C/BV	662 ± 1	94,000	680 ± 1	14.5 ± 0.5	1.53 ± 0.02
iRFP713/C15S/V256C/BV	665 ± 2	66,000	676 ± 2	7.2 ± 0.3	1.31 ± 0.02
iRFP713/PCB	675 ± 1	72,800	700 ± 2	8.5 ± 0.5	0.82 ± 0.03
iRFP713/C15S/PCB	674 ± 1	74,000	698 ± 1	17 ± 1.5	1.24 ± 0.02
iRFP713/V256C/PCB	646 ± 1	77,200	667 ± 1	45 ± 2.0	2.05 ± 0.01
iRFP713/C15S/V256C/PCB	647 ± 1	77,400	666 ± 1	50 ± 2.5	2.12 ± 0.01

^1^ The value is taken from Reference [28].

## Supplementary Materials

Supplementary Materials can be found at https://www.mdpi.com/1422-0067/20/23/6067/s1.

## Abbreviations

BDFP	“Big Dipper constellation” Fluorescent Protein
BV	biliverdin IXα
GAF	cGMP phosphodiesterase/Adenyl cyclase/FhlA domain
GFP	Green Fluorescent Protein
PAS	Per/Arnt/Sim domain
PCB	phycocyanobilin
BphP	bacterial phytochrome
NIR FP	near infrared fluorescent protein
CBCR	cyanobacteriophytochrome
