# Supplementary material for: Near-Infrared Markers based on Bacterial Phytochromes with Phycocyanobilin as a Chromophore
Source: Int J Mol Sci. 2019 Dec 2;20(23):6067. doi: 10.3390/ijms20236067 (PMC6928796; doi:10.3390/ijms20236067)
Supplement: Supplementary file 1 [file ijms-20-06067-s001.pdf]

# Near-infrared markers based on bacterial phytochromes with phycocyanobilin as a chromophore

*Olesya V. Stepanenko, Olga V. Stepanenko, O.G. Shpironok, A.V. Fonin, I.M. Kuznetsova, K.K. Turoverov*

## Supplementary Figures

**Supplementary Figure 1.** SDS-PAGE of iRFP713 in the apoform and in the PCB-bound form.

**Supplementary Figure 2.** Tryptophan fluorescence of the iRFP713 variants assembled with PCB.

**Supplementary Figure 3**. Absorption of iRFP713 assembled with PCB or BV.

**Supplementary Figure 4**. Near-infrared fluorescence of the iRFP713 variants assembled with PCB.

**Supplementary Figure 5**. The near-UV CD spectra of the iRFP713 variants assembled with PCB or BV.

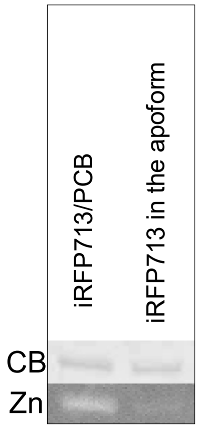


**Supplementary Figure 1. SDS-PAGE of iRFP713 in the apoform and in the PCB-bound form.** The SDS-PAGE of the proteins followed by staining with Coomassie blue (CB) and $ZnCl_2$ (Zn) showed that the PPIX- and BV-bound protein impurities does not significantly contribute to Zn-induced fluorescence of the apoprotein.

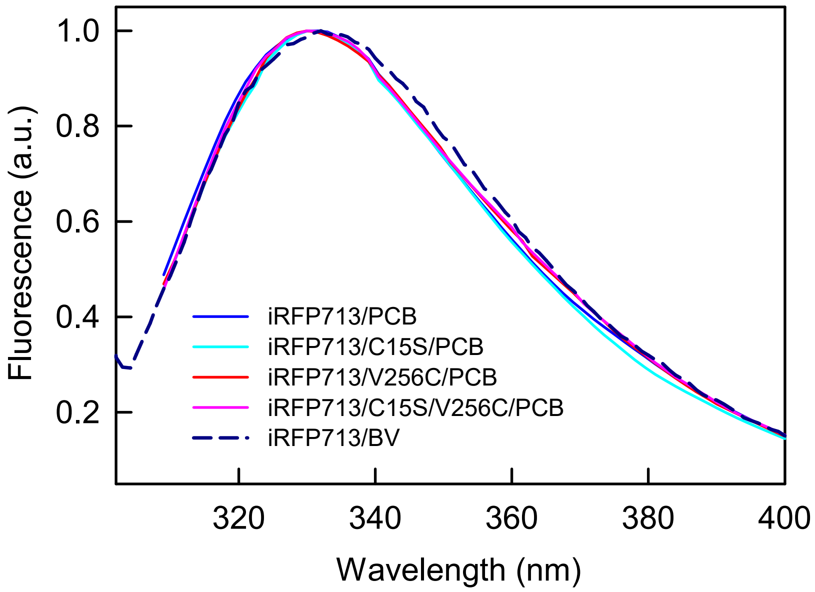


**Supplementary Figure 2. Tryptophan fluorescence of the iRFP713 variants assembled with PCB.** The spectra are normalized to unity at the maximum of fluorescence intensity. The tryptophan fluorescence spectrum of the BV-bound iRFP713 is also shown. The excitation wavelength is 295 nm.

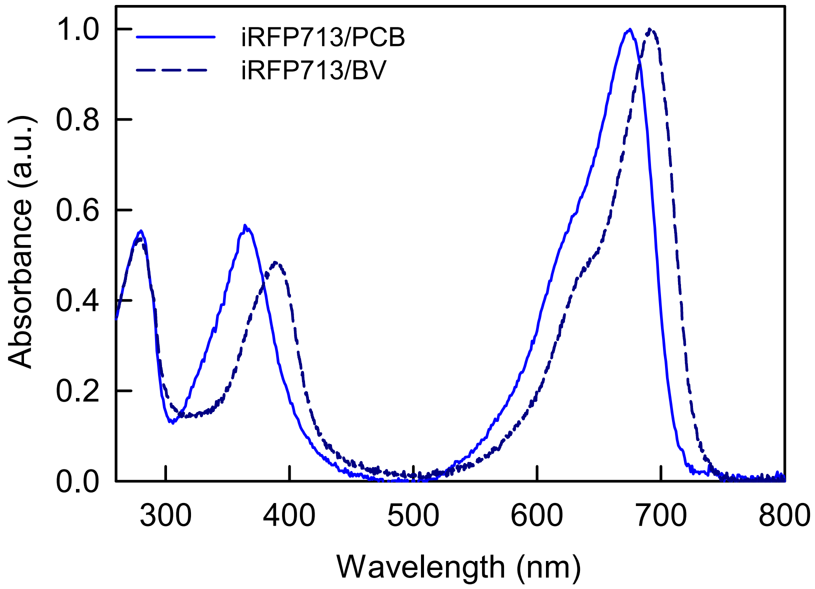


**Supplementary Figure 3. Absorption of iRFP713 assembled with PCB or BV.** The spectra are normalized to unity at the maximum of the Q absorption band of BV or PCB.

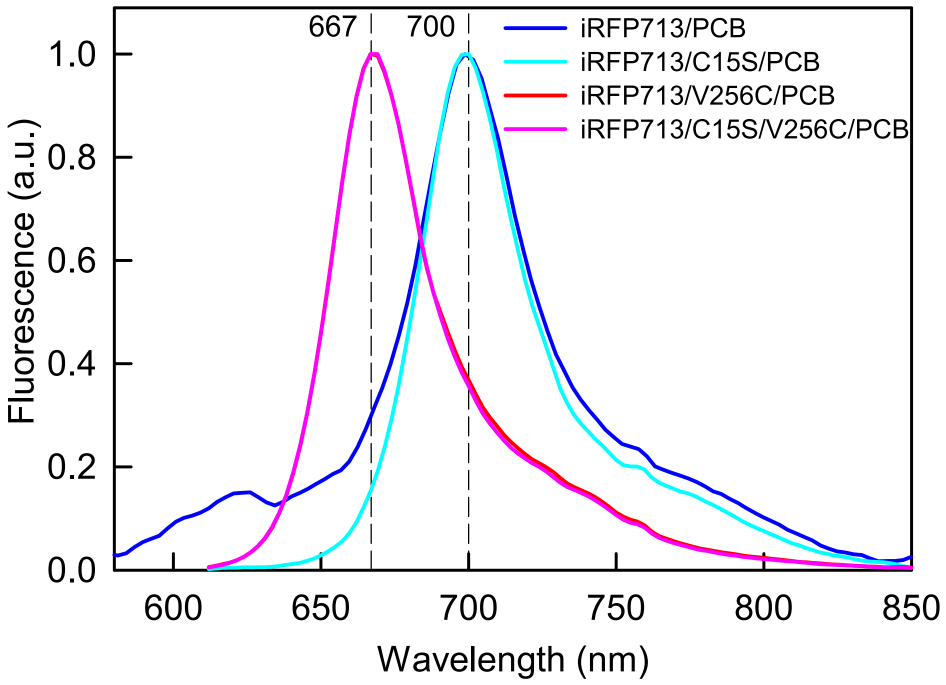


**Supplementary Figure 4. Near-infrared fluorescence of the iRFP713 variants assembled with PCB.** The excitation wavelength is 560 nm.

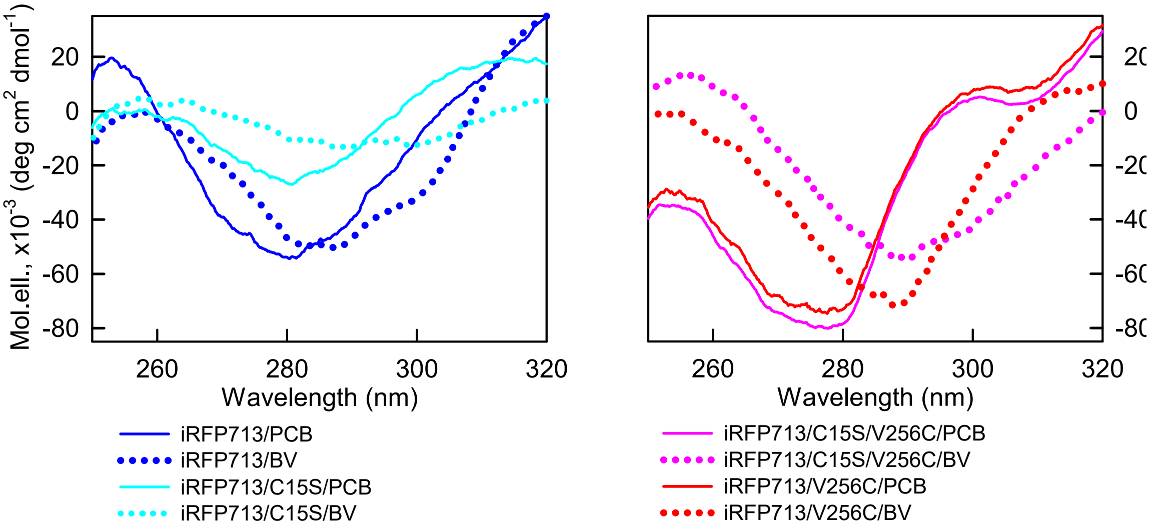


**Supplementary Figure 5. The near-UV CD spectra of the iRFP713 variants assembled with PCB (solid lines) or BV (dotted lines).**
